# 25-Hydroxivitamin D Serum Concentration, Not Free and Bioavailable Vitamin D, Is Associated with Disease Activity in Systemic Lupus Erythematosus Patients

**DOI:** 10.1371/journal.pone.0170323

**Published:** 2017-01-13

**Authors:** Marina Eloi, Daniela Vargas Horvath, João Carlos Ortega, Mônica Simon Prado, Luis Eduardo Coelho Andrade, Vera Lúcia Szejnfeld, Charlles Heldan de Moura Castro

**Affiliations:** Rheumatology Division, Universidade Federal de São Paulo/Escola Paulista de Medicina (Unifesp/ EPM), São Paulo—Brazil; National Institute of Health, ITALY

## Abstract

We aim to evaluate the prevalence of vitamin D deficiency in patients with systemic lupus erythematosus (SLE) and investigate the association between total, free and bioavailable vitamin D serum concentrations and disease activity. Patients with SLE (ACR 1997) consecutively seen at UNIFESP’s outpatient’s clinics had disease activity measured after clinical and laboratory evaluation using SLEDAI (Systemic Lupus Erythematosus Disease Activity Index). 25-hydroxyvitamin D (25(OH)D) serum concentrations measured by chemiluminescence and vitamin D binding protein (DBP) measured by ELISA were used to calculate free and bioavailable vitamin D. Healthy blood donors were used as controls. A total of 142 patients (71.4%) had 25(OH)D serum concentrations below 30 ng/mL. Total 25(OH)D serum concentration was associated with disease activity categorized in 5 continuous groups of SLEDAI. 25(OH)D serum concentrations were higher among patients with SLEDAI 1–5 and lower in those with severe activity (SLEDAI≥20) (p <0.05). On the other hand, no statistically significant difference was observed for DBP, free and bioavailable vitamin D measurements in the disease activity subgroups evaluated. Vitamin D deficiency is highly prevalent among patients with SLE and was associated with higher disease activity. DBP serum level and calculation of free and bioavailable vitamin D were not associated with SLE disease activity.

## Introduction

Systemic lupus erythematosus (SLE) is a chronic multisystem inflammatory autoimmune disease [1]. Several studies have reported that vitamin D deficiency is more prevalent in SLE patients than in the general population [2–6]. One possible explanation for the association between SLE and vitamin D deficiency is the universal recommendation that these patients should avoid sunlight exposure [7]. Moreover, many drugs used in SLE management, such as glucocorticoids and hydroxychloroquine, may interfere with vitamin D metabolism and 25-hidroxyvitamin D (25(OH)D) serum levels [8]. It has also been suggested that vitamin D deficiency might be a risk factor for the development of the disease, although vitamin D intake was not associated with the risk of SLE development in a prospective study [9].

Vitamin D deficiency seems to be associated with immunological abnormalities in SLE. Some *in vitro* evidence implies that vitamin D modulates the differentiation and activity of T and B-lymphocytes and, therefore, the production of autoantibodies [10]. On the other hand, the association between vitamin D serum concentration with disease activity and prognosis in SLE remains controversial. In spite of some studies with interesting results [11], the literature still lacks of convincing proof demonstrating that vitamin D supplementation in patients with SLE can modify disease progression.

An association between high disease activity in SLE with low vitamin D serum concentrations has been reported, but these results are controversial [12]. In children and adolescents with SLE no correlation was found between glucocorticoid or hydroxychloroquine use, cumulative dose of glucocorticoid and vitamin D serum concentration [13]. Other authors have also failed to demonstrate association between vitamin D and SLE disease activity [14]. An inverse correlation between vitamin D serum concentration and disease activity in children with juvenile SLE has been reported [15]. In spite of such association, a causal relationship between vitamin D serum concentration and disease activity in SLE patients could not be established [16].

Part of the disagreement regarding a potential role for vitamin D in SLE disease activity [9,12,15,13] may be due to the fact that these studies have not evaluated the free and bioavailable fractions of vitamin D. It is possible that bioavailable and free vitamin D might be more reliable biological markers of vitamin D *status* than the total 25(OH)D serum concentration measurement. In the present study we assess the prevalence of vitamin D deficiency in a cohort of patients with SLE and examine the association between total, free and bioavailable vitamin D serum measurements with disease activity.

## Patients and Methods

The study included a total of 199 patients diagnosed with SLE according to the American College of Rheumatology (ACR) 1997 classification criteria [17]. All participants agreed to participate in the study. The inclusion of patients from the Rheumatology Outpatient Clinics at UNIFESP, São Paulo–Brazil, was done by convenience in consecutive clinical appointments from February 2014 to January 2016. A total of 350 patients were evaluated and 199 met inclusion criteria described below.

Patients with overlapping findings with other systemic autoimmune diseases, rituximab use or plasmapheresis six months before or during the course of the study, bone marrow transplantation, acquired immunodeficiency syndrome (AIDS), neoplastic disease (except basal cell carcinoma) and common variable immunodeficiency were excluded. Use of vitamin D supplements was not an exclusion criterion.

All patients were contacted for clarification on the nature of the study and gave written informed consent to participate in the study. Minors/children were not included in the present study. The UNIFESP’s Ethics Committee approved the study protocol.

Anthropometric, demographic and clinical data collected from the electronic medical charts or clinical interview included age, sex, race, weight, height, disease duration and medication in use. Disease activity was measured using Systemic Lupus Erythematosus Disease Activity Index (SLEDAI) at the time that blood samples were collected. Nephritis was assessed taking into account the presence of the following parameters: urine protein-to-creatinine ratio (or 24-hour urine protein) representing 500 mg protein/24 hours or red blood cell casts or renal biopsy with abnormalities suggestive of lupus nephritis.

Potential differences in vitamin D serum concentrations in this sample of patients with SLE were examined using the categorization of disease activity in 5 distinct levels: Inactive disease (SLEDAI = 0), light activity (SLEDAI 1 to 5), moderate activity (SLEDAI 6 to 10), high activity (SLEDAI 11 to 19) and severe activity (≥20 SLEDAI), as previously published [18]. These categories of SLE disease activity have been established according to the relative risk of death.

A total of 150 healthy volunteers selected among blood donors without SLE were used as control group.

Blood samples were collected by intravenous puncture and serum aliquots were stored at -80°C for the biochemical analyses described below.

25(OH)D serum measurements were performed on a Siemens ADVIA Centaur apparatus using chemiluminescence technique. Total 25(OH)D measurement coefficient of variation using this method is 11.7%.

Vitamin D binding protein (DBP) was measured in SLE patients by monoclonal antibody ELISA (Cloud-Clone Corp. kit—USCN Life Science Inc.) using standard technique. The coefficient of variation (CV%) for DBP measurement is 4.8%. DBP measurements in the study were considered only when duplicate measures were available. In 39 SLE samples DBP was not performed in duplicate and so was not used in the analysis.

DBP measurements were used to calculate free and bioavailable vitamin D according to previously developed equation [19] further adapted [20]. Bioavailable vitamin D was calculated as the sum of free vitamin D with 25(OH)D bound to albumin.

Bone densitometry data was available for a total of 107 patients included in the present analysis. Bone mineral density (BMD) measurements were performed at the lumbar spine (L1-L4) and proximal femur (neck and total hip) using dual energy X-ray absorptiometry (DXA) (DPX MD +, GE-Lunar, Madison, WI, USA). The coefficient of variation for BMD measurement was 1.5% and 2% at lumbar spine and total hip, respectively.

### Statistical Analysis

Descriptive statistics (mean, standard deviation for quantitative variables, and frequency and percentage for categorical variables) was used to characterize patients and their groups. Quantitative variables were compared between groups of independent samples using Student's *t* test for normally distributed variables and Mann-Whitney test for variables with non-normal distribution. For prospective analyses (dependent samples), quantitative variables were compared using ANOVA. Categorical variables were analyzed using Chi-square test with Bonferroni correction for multiple comparisons. Statistical analyzes were performed using SPSS software version 17.0 (Chicago, IL). Significance level was set as p <0.05.

## Results

A total of 199 consecutive patients with SLE were included in the study. Demographic, clinical and laboratorial data for these patients and their healthy controls are shown in Table 1. SLE patients were 37.2 ± 11.1 years old (range 26 to 48 years old) and mainly women (96%). 25(OH)D serum concentrations were significantly lower in SLE patients as compared to healthy controls matched for age and BMI. Vitamin D deficiency (25(OH)D lower than 20 ng/mL) and insufficiency (25(OH)D lower than 30 ng/ml) were highly prevalent in SLE patients. A total of 142 SLE patients (71.4%) had 25(OH)D serum concentrations below 30 ng/mL, significantly higher than that seen for healthy controls (p<0,001). Only 57 SLE patients (28.6%) had 25(OH)D serum concentrations exceeding 30 ng/mL (vitamin D sufficiency). DBP measurements were available for 160 patients with SLE. Free and bioavailable vitamin D was calculated in those patients.

**Table 1:** Demographic, anthropometric, clinical and laboratorial parameters in Systemic Lupus Erythematosus patients and their healthy controls.

	Healthy controls (N = 150)	SLE (N = 199)	*p*
**Sex,N(%)**			
Women	135(90)	191(96)	0.231
Men	15(10)	8(4)	
**Race, N(%)**			
White	93(62)	111(55.7)	0.238
Mixed	52(35)	81(40.7)	
Black	5(3)	7(3.6)	
**Age** (years)	36.5±10.9	37.2±11.1	0.423
**Weight** (kg)	70.2±15.2	69.8±16.2	0.217
**Height** (m)	1.60±0.08	1.59±0.08	0.208
**BMI** (kg/m^2^)	27.4±5.5	27.7±6.0	0.880
**Disease duration** (years)	-	9.7±7.2	**-**
**25(OH)D** (ng/mL)	28.79±7.82	26±7.93	**0.009**
**≥30 ng/mL (%)**	33.96	28.64	**<0.001**^**&**^
**20–30 ng/mL (%)**	59.43	47.24	
**<20 ng/mL (%)**	6.60	24.12	
**DBP** (ng/mL)*		1.91±0.89	
**Free vitamin D** (pg/mL)*		11.10±5.44	
**Bioavailable vitamin D** (ng/mL)*		4.32±2.12	

*N = 160

^**&**^ ANOVA.

Vitamin D serum concentrations according to SLEDAI categories are shown in Table 2.

**Table 2:** Serum 25(OH)D (ng/mL), D binding protein (DBP) (ng/mL), free (pg/mL) and bioavailable (ng/mL) vitamin D in Systemic Lupus Erythematosus patients, according to disease activity measured by Systemic Lupus Erythematosus Disease Activity Index (SLEDAI).

	Disease Activity				
	InactiveSLEDAI 0(N = 30)	MildSLEDAI 1-5(N = 69)	ModerateSLEDAI 6-10(N = 42)	HighSLEDAI 11-19(N = 40)	SevereSLEDAI≥20(N = 18)
25(OH)D*	28.45±6.83	27.70±7.81	25.16±6.38	26.98±9.93	22.22±6.96**
**DBP**	1.80±0.92(N = 27)	1.89±0.70(N = 61)	1.79±0.54(N = 33)	2.14±1.42(N = 29)	1.97±0.87(N = 9)
**Free vitamin D**	11.29±7.24(N = 27)	11.67±5.12(N = 61)	10.79±3.84(N = 33)	10.86±5.85(N = 29)	8.54±5.20(N = 9)
**Bioavailablevitamin D**	4.39±2.82(N = 27)	4.55±2.00(N = 61)	4.20±1.50(N = 33)	4.23±2.28(N = 29)	3.32±2.02(N = 9)

*p = 0.042 (ANOVA)

**p<0.001 versus inactive and light activity (Tukey).

A statistically significant difference between 25(OH)D serum concentrations was observed between the categories of disease activity (p = 0.042; ANOVA, Table 2). It was demonstrated a statistically significant difference between 25(OH)D values between inactive disease (SLEDAI = 0) or light activity (SLEDAI 1–5) and severe activity (SLEDAI≥20) (p<0.001; Tukey test). On the other hand, mean values for DBP, free and bioavailable vitamin D did not differ significantly between the categories of disease activity.

The association between vitamin D serum concentration and disease activity in our sample was also tested by assessing the *status* of vitamin D, as defined by the US Endocrine Society [21]: deficiency (25(OH)D lower than 20 ng/mL), insufficiency (25(OH)D between 20 and 30 ng/mL) and sufficiency (25(OH)D ≥ 30 ng/mL). Table 3 shows the prevalence of different *status* of vitamin D according to disease activity. Since there was no statistically significant difference between the *statuses* insufficiency and deficiency, these two strata were analyzed together and compared to the sufficiency *status*.

**Table 3:** Vitamin D *status* (deficiency: 25(OH)D lower than 20 ng/mL; insufficiency: 25(OH)D between 20 and 30 ng/mL; and sufficiency (25(OH)D ≥ 30 ng/mL) in Systemic Lupus Erythematosus patients, according to disease activity measured by Systemic Lupus Erythematosus Disease Activity Index (SLEDAI).

	SLEDAI				
	0(N = 30)	1-5(N = 69)	6-10(N = 42)	11-19(N = 40)	≥20(N = 18)
25(OH)D < 30 ng/mL, N (%)*	15 (11.5)	40 (30.6)^#^	34 (25.9)	25 (19.1)	17 (12.9)^&^
25(OH)D ≥ 30 ng/mL, N (%)*	15 (22.1)	29 (42.6)^#^	8 (11.8)	15 (22.1)	1 (1.4)^&^

*p = 0.001 (Chi-square)

^#^ p = 0.003

^&^ p = 0.026.

As shown in Table 3, the two groups (25(OH)D < 30 ng/mL *versus* ≥ 30 ng/mL) differ significantly in the proportions of disease activity (p = 0.001). The proportion of patients with vitamin D sufficiency (25(OH)D ≥ 30 ng/mL) is significantly higher in the groups SLEDAI 0 and 1–5 when compared to 25(OH)D values below 30 ng/mL (p = 0.003). In patients with severe activity (SLEDAI ≥20) we observed the contrary: the proportion of patients with 25(OH)D serum concentration lower than 30 ng/mL was significantly higher as compared to values ≥ 30 ng/mL (p = 0.026).

Potential associations between vitamin D *status* and season, body mass index (BMI), Bone Mineral Density (BMD) and use of medication were also tested. There was no statistically significant difference in the mean serum concentrations of 25(OH)D (p = 0.179), free and bioavailable vitamin D (p = 0.441) between the different seasons (ANOVA) (S1 Table).

25(OH)D serum concentrations were not significantly associated with disease duration or BMI. Both free and bioavailable vitamin D was also not associated with those variables.

Spine and hip BMD did not correlate significantly with 25(OH)D, free or bioavailable vitamin D serum concentrations. Moreover, BMD did not differ significantly among the various vitamin D *statuses* (S2 Table).

Possible associations between 25(OH)D serum concentration and the use of medications were considered. Table 4 shows that vitamin D serum levels were not associated significantly with the use of medications commonly used for management of SLE. Only the use of cyclophosphamide was associated with lower 25(OH)D serum concentrations (p = 0.020). This association was not confirmed when the analysis was performed according to the statuses of vitamin D (sufficiency or insufficiency/deficiency) (p = 0.065). A total of 74 patients were in use of cholecalciferol (400 to 1000 IU/day). The data demonstrate that patients taking cholecalciferol had 25(OH)D serum concentrations higher than the others with no supplementation (p<0.001). Similarly, the prevalence of vitamin D deficiency or insufficiency was significantly higher among patients without supplementation as compared to patients receiving cholecalciferol.

**Table 4:** Serum 25(OH)D (mean ± SD) and vitamin D status (deficiency: 25(OH)D lower than 20 ng/mL; insufficiency: 25(OH)D between 20 and 30 ng/mL; and sufficiency (25(OH)D ≥ 30 ng/mL) in Systemic Lupus Erythematosus patients, according to medication use.

		25(OH)D(ng/mL)	Deficiency + InsufficiencyN (%)	SufficiencyN (%)
**Prednisone**	Yes	26.4±7.9	53 (69.7)	23 (30.3)
	No	25.7±8.0	89 (72.4)	34 (27.6)
**Hydroxychloroquine**	Yes	25.6 ±7.3	111 (73.0)	41 (27.0)
	No	27.3±9.5	31 (66.0)	16 (34.0)
**Azathioprine**	Yes	26.2±8.3	28 (65.1)	15 (34.9)
	No	25.9±7.8	114 (73.1)	42 (26.9)
**Mycophenolate**	Yes	27.1±9.1	19 (70.4)	8 (29.6)
	No	25.8±7.7	123 (71.5)	49 (28.5)
**Cyclophosphamide**	Yes	21.3±5.2	13 (92.9)	1 (7.1)
	No	26.4±8.0	129 (69.7)	56 (30.3)
**Cyclosporine**	Yes	29.1±2.6	2 (66.7)	1 (33.3)
	No	26.0±8.0	140 (71.4)	56 (28.6)
**Methylprednisolone**	Yes	23.7±5.7	11 (84.6)	2 (15.4)
	No	26.2±8.1	131 (70.4)	55 (29.6)
**Methotrexate**	Yes	27. 3±7.0	17 (65.4)	9 (34.6)
	No	25.8±8.1	125 (72.3)	48 (27.7)
**Cholecalciferol**	Yes	27.9±7.8*	26 (35.1)	48 (64.8)
** **	No	24.9±7.7	94 (75.2)	31(24.8)

*p<0.001.

## Discussion

In the present study we investigated the association between vitamin D serum concentration, its free and bioavailable fractions and disease activity in SLE patients. We have observed a very high prevalence of vitamin D deficiency and insufficiency among SLE patients. Only 28.6% of the patients were vitamin D sufficient (serum 25(OH)D ≥ 30 ng/mL). Vitamin D deficiency was significantly more frequent in SLE patients than in healthy controls. Vitamin D deficiency is then more prevalence in SLE patients as compared to the Brazilian general population without SLE [22–25]. Significantly lower prevalence of vitamin D deficiency has been reported in SLE patients in Brazil [14]. We have also found that disease activity in SLE was associated with lower 25(OH)D serum concentration. On the other hand, measuring DBP with a monoclonal ELISA in this clinical setting has not added information to the *status* of vitamin D: both free and bioavailable fractions of 25(OH)D did not differ between the various categories of SLE disease activity.

Some cross-sectional studies have shown an inverse correlation between vitamin D serum concentration and disease activity in SLE patients [5,6,12]. On the other hand, in 159 SLE patients in the city of Rio de Janeiro, 25(OH)D serum concentration measured by high performance liquid chromatography (HPLC) did not correlate with activity or duration of the disease, sunlight exposure, vitamin D supplementation, glucocorticoid use or renal function [14].

The importance of free and bioavailable vitamin D has been tested in other clinical scenarios. Low 25(OH)D serum levels have been associated with high risk of multiple sclerosis [26]. Free and bioavailable vitamin D did not differ between multiple sclerosis patients and their healthy controls [27]. In pediatric patients with chronic kidney disease, both free and bioavailable vitamin D were significantly lower than in healthy controls [28]. Additionally, it was demonstrated that vitamin D2 (ergocalciferol) or D3 (cholecalciferol) supplementation in hip fracture patients resulted in no significant differences in free and bioavailable vitamin D [29].

It was recently demonstrated that DBP measurements using monoclonal ELISA are biased by DBP genotype and race [30]. DBP values varied significantly when measured by monoclonal or polyclonal ELISA or tandem mass spectrometry and that had implication on free and bioavailable vitamin D calculations. Based on those findings, one could speculate that measuring DBP with polyclonal or tandem mass spectrometry would render different results and that is a limitation of our present study.

Vitamin D serum concentrations did not correlate with the seasons in sample of patients with SLE, unlike what has been observed in the general population [21]. This is probably the result of the universal recommendation that SLE patients should use continuous photo protection and avoid sun exposure, regardless of the season.

In this study, vitamin D supplementation was associated with increased 25(OH)D serum concentrations. The reported doses of 400 to 1000 IU/day do not seem to be sufficient to ensure adequate levels of vitamin D. About 35% of patients using cholecalciferol still presented vitamin D deficiency or insufficiency, suggesting that higher doses are required for proper supplementation in these patients.

Due to its cross-sectional nature, our study cannot establish a causal relationship between vitamin D serum concentration and disease activity in SLE patients. If low vitamin D serum concentrations are causal co-factor in the immunological disturbances that characterize SLE or if, on the contrary, the inflammatory disease process and low sun exposure causes reduction in vitamin D serum concentrations will still require further studies [26]. It is also possible that the sample size may have affected our results. Perhaps larger samples may establish better relationship between vitamin D measurements and disease activity in SLE patients.

In conclusion, we have demonstrated a very high frequency of vitamin D deficiency and insufficiency among patients with SLE. In this clinical scenario, disease activity was associated with lower serum concentrations of 25(OH)D. DBP measurements with monoclonal ELISA and free and bioavailable vitamin D calculations did not differ among different categories of SLE disease activity. Prospective studies are needed to investigate and establish a potential causal relationship between vitamin D *status* and disease activity in SLE.

## Supporting Information

S1 Table 25(OH)D, free and bioavailable vitamin D (mean ± standard deviation) serum concentration in Systemic Lupus Erythematosus patients, according to season. (DOCX)

S2 Table Bone mineral density (BMD, g/cm2) in Systemic Lupus Erythematosus patients, according to vitamin D status (deficiency: 25(OH)D lower than 20 ng/mL; insufficiency: 25(OH)D between 20 and 30 ng/mL; and sufficiency (25(OH)D ≥ 30 ng/mL). (DOCX)
