# Supplementary material for: 25-Hydroxivitamin D Serum Concentration, Not Free and Bioavailable Vitamin D, Is Associated with Disease Activity in Systemic Lupus Erythematosus Patients
Source: PLoS One. 2017 Jan 13;12(1):e0170323. doi: 10.1371/journal.pone.0170323 (PMC5234837; doi:10.1371/journal.pone.0170323)
Supplement: S1 Table — (DOCX) [file pone.0170323.s001.docx]

**S1 Table. 25(OH)D, free and bioavailable vitamin D (mean ± standard deviation) serum concentration in Systemic Lupus Erythematosus patients, according to season.**

|  | **Summer** | **Fall** | **Winter** | **Spring** |
| --- | --- | --- | --- | --- |
| **25(OH)D (**ng/mL) | 26.48 ± 8.74 | 27.30 ± 7.27 | 23.82 ± 7.38 | 26.13 ± 8.17 |
| **Free vitamin D** (pg/mL) | 11.32 ± 6.81 | 11.74 ± 4.09 | 9.86 ± 5.25 | 11.40 ± 5.61 |
| **Bioavailable vitamin D** (ng/mL) | 4.41 ± 2.65 | 4.57 ± 1.59 | 3.84 ± 2.04 | 4.44 ± 2.19 |
