# Supplementary material for: 25-Hydroxivitamin D Serum Concentration, Not Free and Bioavailable Vitamin D, Is Associated with Disease Activity in Systemic Lupus Erythematosus Patients
Source: PLoS One. 2017 Jan 13;12(1):e0170323. doi: 10.1371/journal.pone.0170323 (PMC5234837; doi:10.1371/journal.pone.0170323)
Supplement: S2 Table — (DOCX) [file pone.0170323.s002.docx]

**S2 Table: Bone mineral density (BMD, g/cm^2^) in Systemic Lupus Erythematosus patients, according to vitamin D status (deficiency: 25(OH)D lower than 20 ng/mL; insufficiency: 25(OH)D between 20 and 30 ng/mL; and sufficiency (25(OH)D ≥ 30 ng/mL).**

|  | **Deficiency + Insufficiency** | **Sufficiency** |
| --- | --- | --- |
| **Lumbar spine L1L4** |  |  |
| **BMD** | 1.09±0.17 | 1.04±0.19 |
| **T-score** | (0.7)±1.35 | (1.0)±1.44 |
| **Z-score** | (0.64)±1.22 | (0.94)±1.37 |
| **Neck** |  |  |
| **BMD** | 0.92±0.17 | 0.93±0.15 |
| **T-score** | (0.56)±1.2 | (0.25)±1.02 |
| **Z-score** | (0.31)±1.06 | (0.08)±1.14 |
| **Total femur** |  |  |
| **BMD** | 0.95±0.16 | 0.97±0.14 |
| **T-score** | (0.41)±1.30 | (0.11)±1.14 |
| **Z-score** | (0.32)±1.19 | (0.05)±1.18 |
